# Preventing and Lessening Exacerbations of Asthma in School-aged children Associated with a New Term (PLEASANT): Recruiting Primary Care Research Sites–the PLEASANT experience

**DOI:** 10.1038/npjpcrm.2015.66

**Published:** 2015-11-12

**Authors:** Michelle J Horspool, Steven A Julious, Cara Mooney, Robin May, Ben Sully, W Henry Smithson

**Affiliations:** 1 School of Health and Related Research, University of Sheffield, Sheffield, UK; 2 Clinical Practice Research Datalink, The Medicines and Healthcare products Regulatory Agency, London, UK; 3 Department of General Practice, University College Cork, Cork, Ireland

## Abstract

**Background:**

Recruitment of general practices and their patients into research studies is frequently reported as a challenge. The Preventing and Lessening Exacerbations of Asthma in School-aged children Associated with a New Term (PLEASANT) trial recruited 142 general practices, across England and Wales and delivered the study intervention to time and target.

**Aims:**

To describe the process of recruitment used within the cluster randomised PLEASANT trial and present results on factors that influenced recruitment.

**Methods:**

Data were collected on the number of and types of contact used to gain expression of interest and subsequent randomisation into the PLEASANT trial. Practice size and previous research experience were also collected.

**Results:**

The mean number of contacts required to gain expression of interest were *m*=3.01 (s.d. 1.6) and total number of contacts from initial invitation to randomisation *m*=6.8 (s.d. 3.5). Previous randomised controlled trial involvement (hazard ratio (HR)=1.81 (confidence interval (CI) 95%, 1.55–2.11) *P*<0.001) and number of studies a practice had previously engaged in (odds ratio (OR) 1.91 (CI 95%, (1.52–2.42)) *P*<0.001), significantly influenced whether a practice would participate in PLEASANT. Practice size was not a significant deciding factor (OR=1.04 (95% CI 0.99–1.08) *P*=0.137).

**Conclusions:**

Recruitment to time and target can be achieved in general practice. The amount of resource required for site recruitment should not, however, be underestimated and multiple strategies for contacting practices should be considered. General practitioners with more research experience are more likely to participate in studies.

## Introduction

Recruitment of general practices (GPs) and their patients into research studies is often reported as a challenge.^1–3^ This difficulty in recruitment has a negative impact on recruitment targets, delivery to pre-planned timelines and associated costs, which can have implications for the validity of the findings and how representative they may be.^2,4^

A survey of UK primary care studies reported that 56% of studies extended the recruitment period, 44% increased the number of sites, and 31% sought additional funding in order to complete the trial.^5^ The additional resource sometimes required to complete studies in primary care can discourage both researchers and funders. Although there has been published guidance on the principles of improving recruitment in primary care^2^ and information from systematic reviews of recruitment in clinical trials generally,^6^ the lack of evidenced-based recommendations makes targeting interventions for improving recruitment difficult. As such the process of recruitment remains complex and challenging.

Randomised control trials that recruit from GPs and achieve recruitment targets can add to the evidence base by reporting the process of recruitment and characteristics of the participating primary care sites. The PLEASANT trial (Preventing and Lessening the Exacerbation of Asthma in School-aged children Associated with a New Term) recruited from GPs to time and exceeded target. This paper will report the experience of the PLEASANT trial and factors that contributed to practices expressing an interest and actual participation in the trial. Characteristics of recruited practices were analysed to identify predictors of successful participation.

### Background to the PLEASANT trial

In the United Kingdom, there is a significant increase in the number of unscheduled visits to the doctor, by school-aged children with asthma associated with the return back to school in September after the summer holidays. This increase is preceded by a drop in the number of asthma-related prescriptions administered in August.^7^ It is possible therefore that children might not be taking their medication as prescribed before the viral challenge they meet on return to school and from picking up infections that might affect their asthma.

PLEASANT is a cluster randomised controlled trial (cRCT) that examines whether a brief intervention delivered by the GP and the practice to the parents of school-aged children with asthma, at the start of the summer holidays, would reduce the number of unscheduled medical contacts following return to school in September of the same year.

The trial recruited 142 GPs (70 intervention and 72 control) throughout England and Wales. The target population was school children aged between 4 and 16 years at 1 September 2013 with a coded diagnosis of asthma who had been prescribed asthma medication in the previous 12 months. The intervention (a letter sent from the practitioner signed by a GP to parents/carers of children with asthma reminding them to maintain their child’s medication and collect a prescription if they are running low) was delivered at the start of the school summer holidays w/c 29 July 2013. Docmail (a secure web-based postal service^8^) was encouraged as a vehicle for sending the intervention to reduce the research burden to sites. This also facilitated monitoring the timing of the intervention and the numbers of letters sent. The primary outcome was the proportion of patients who have an unscheduled medical contact in September 2013 (see Horspool *et al.* ^9^ for full details of the trial).

## Methods

Recruitment to the PLEASANT trial took place over a 7-month period from January 2013 to July 2013. This was a fixed recruitment period, with no possibility of extension due to the timing of the intervention, with a target recruitment sample size of 140. GPs in England and Wales, linked to the Clinical Practice Research Datalink (CPRD),^10^ were invited to participate in the PLEASANT trial (*n*=433). CPRD is a computerised database that is able to access pseudonymised routine medical records from primary care—the database holds information on all medical contacts and records the type of contact based on system codes. CPRD was used due to the amount of follow-up data required—the study collected all medical contacts—for children diagnosed with asthma, over a 2-year period (12 months pre- and post intervention).

The National Institute for Health Research, Primary Care Research Networks (PCRN) also advertised the study and supported recruitment. The eligibility criteria were that had to be using Vision software system (London, UK) (as this is the software that was linked to CPRD at the time), and had to be willing to join CPRD and supply data if they were not already.

### Recruitment process

A staged recruitment strategy was undertaken by CPRD, which consisted of repeat invitations by post or email to all 433 practices from England and Wales contributing to the database. Invitations (which included a detailed study information sheet and an expression of interest (EOI) form developed by the study team—details can be found on the PLEASANT website^11^) were sent to either the practice research lead (usually GP) and/or practice manager depending on the practices’ preferred contact person given when they joined CPRD. The first contact was by postal invitation. Practices who did not respond to the postal invitation were sent a second invitation via email. A further two emails were sent to non-responding practices followed by a final, posted, letter of invitation. A final round of recruitment, to boost uptake towards the end of the recruitment period, was a personal telephone call to the GP, practice manager or lead research contact at the sites. The telephone calls were conducted either by CPRD or by members of the study team including a research assistant and trial manager who are based in the Sheffield Clinical Trials Research Unit (CTRU). Where practice access proved difficult, the practices were phoned by the GP member of the team.

Practices were asked to return the completed EOI (information included practice name and address, name/email of the practice research lead, practice manager or other study contact, practice information technology system and Clinical Commissioning Group area) to either the CPRD or directly to the study team. The research assistant, at the University of Sheffield, then attempted to contact the practice via telephone within 2 working days of receiving the EOI to discuss the study further and arrange a telephone study set-up meeting. Once the set-up meeting had been completed and the practice gave verbal consent to participate, they were randomised, by a CTRU statistician within 48 hours and informed via email of their allocation. Practices were asked to acknowledge receipt of allocation. Communication from receiving the EOI to randomisation was either by telephone or email.

### Data collection (practice specific)

Data were collected on the number of times a practice was contacted and the type of contact (postal, email or telephone). From information available from CPRD records, data were also collected on practice size, and on the number and type of previous studies a practice had participated in to examine what impact this may have had on participation in PLEASANT.

### Statistical analysis

The binary responses of EOI and whether or not a practice was randomised were analysed by logistic regression. The explanatory variables: number previous studies—in total and broken down by number of individual or cluster randomised RCT—and practice size were individually analysed against the outcomes. The survival-type responses time to EOI and time to randomisation were analysed by Kaplan–Meier survival plots and Cox regression for the same explanatory variables. For the survival end points, censoring times were defined as the last time a practice was contacted by reminder letter, email or telephone call.

## Results

**Figure 1:**
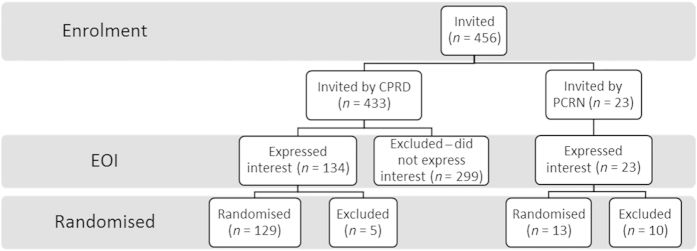
Practice recruitment.

The flow of practices through the full PLEASANT trial is shown in Figure 1. A total of 433 practices were invited to the study by the CPRD, 157 EOIs were received with 142 randomised in total (including those recruited by PCRN *n*=13). Practices were excluded if they had recently changed computer system (*n*=5) or were not willing to join CPRD (*n*=10 PCRN recruited practices). Information relating to the recruitment process used by PCRN to gain EOI is not available; therefore, those practices (*n*=13) are excluded from the remaining analysis and only those practices recruited via CPRD are included in the results (*n*=129).

**Figure 2:**
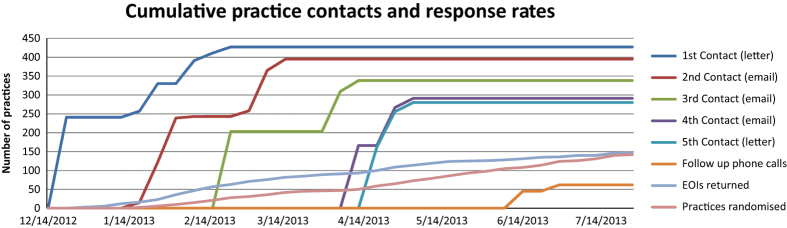
Cumulative practice contacts and response rates.

The number of contacts from initial postal invitation to EOI was *m*=3.01 (s.d. 1.6), from EOI to randomisation *m*=4.4 (s.d. 3.2) and total number of contacts from initial invitation to randomisation *m*=6.8 (s.d. 3.5). Figure 2 shows the timing and type of contacts and the cumulative response rates to EOI and randomisation.

**Figure 3:**
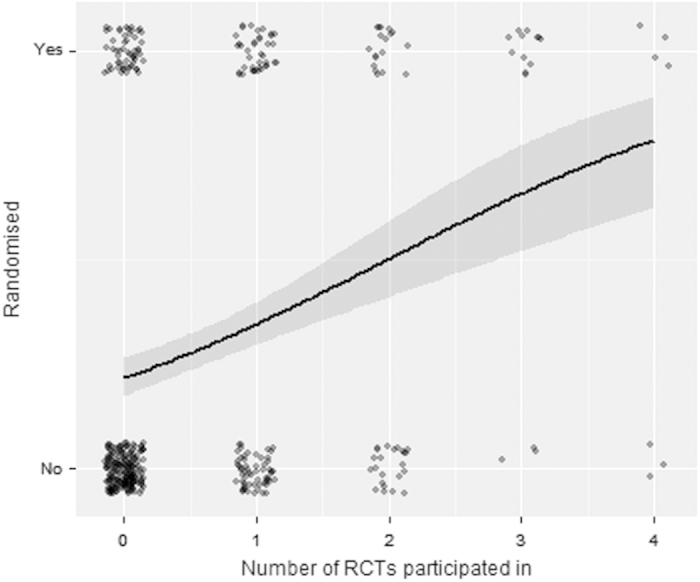
Randomisation and number of previous RCTs.

Logistic regression analysis was undertaken to assess the impact of previous study participation with the outcome as randomisation status and number of previous RCTs a practice had participated in as the explanatory variables (Figure 3). The corresponding odds ratios, for the explanatory variables, are found in Table 1: all models were highly significant with *P*<0.001. For the model fitted to investigate whether the explanatory variable practice size, there was no evidence of effect (*P*=0.137). Table 1 also shows the results of using EOI status as the outcome. These results infer more of an effect for practices who had previously participated in RCTs.

Each of the odds ratios is an odds ratio per increase in the number of trials. Thus, the odds ratio of being in the study for practices who have done one cluster trial compared with those who have done none from the model is 2.42. Likewise, the odds ratio for those who did two cluster trials compared with those who did one is also 2.42. Although the odds ratio for those who did two compared with those who did none from the model is 2.42×2.42=5.86.

**Figure 4:**
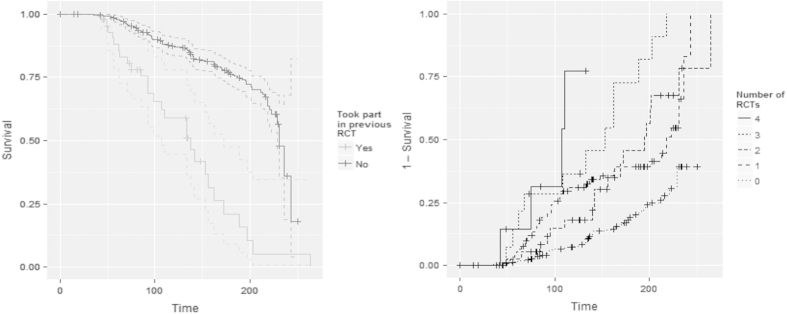
Time to randomisation by previous participation in RCTs and number of RCTs.

Kaplan–Meier plots for time to randomisation broken down by whether a practice had previously participated in a trial and the number of trials are shown in Figure 4 where randomised status was the event and time to randomisation used as the censoring times. As well as whether a practice had undertaken a trial previously, the total number of RCTs seemed to be a significant factor in participation.

Hazard ratios from corresponding Cox regression models are given in Table 2. The results were similar to the logistic regression analysis. All models and variables were highly significant (*P*<0.001) with the exception of practice size, which seemed to not affect the time to randomisation of a practice.

It is more common for a survival analysis to be undertaken on an undesirable outcome, which we wish to prevent—such as mortality—and not an outcome we wish to achieve such as here—recruitment into the study. We did a plot of the log of the negative log of the cumulative hazards to investigate the proportional hazards of the model. There is an initial period at the start of the study—evident in Figure 4—when the assumptions may not be so strong but after this point the assumptions of proportional hazards seem to hold.

## Discussion

### Main findings

Recruitment to time and target can be achieved in primary care trials though it is important to consider the amount of resource that is required. In the PLEASANT study, there was a full-time research assistant within the CTRU, a trial support administrator within CPRD, as well as a trial manager and a clinically active academic GP co-applicant, all of whom gave input to recruiting sites during the 7-month recruitment period. Most of the practices invited where part of the CPRD who have knowledge of the practices and as such judgements were made about the type and amount of invitations made to sites. Giving consideration to the importance of time, resource and researcher effort required for successful recruitment has been reported in other studies that also used intense research strategies, similar to PLEASANT, to achieve recruitment targets.^12–15^

Analysis also suggests having more research experience improves participation and response rates to EOI and randomisation.

### Interpretation of findings in relation to previously published work

Practice list size has been reported as significant with studies reporting larger practices were more likely to participate in research studies.^16,17^ However, Goodyear-Smith^18^ found that larger practices were harder to recruit and, as with PLEASANT, Down^19^ suggests that practice size was not a significant factor in predicting participation rates.

Other demographic factors previously reported as increasing the likelihood of practice participation include practice involvement in GP training^3,20^ higher Quality and Outcome Framework scores,^19^ number of GP partners,^21^ whether sites are training practices and the numbers of GPs at each practice. However, these variables were not available from CPRD, so we are unable to state whether any of these may have had an impact in recruitment to PLEASANT.

### Strengths and limitations of this study

The main strength of the PLEASANT trial was ease of the intervention and data collection. On the basis of previous literature, the trial was designed to reduce the research burden on practices and as such there was minimal activity required from the site; patient identification was done by CPRD (based on agreed diagnostic codes) and Docmail was used for posting the intervention. Communication with sites was maintained throughout the recruitment period that helped retention and ensured the intervention went out per protocol. There was no individual patient follow-up, or data collection required from sites as this was collected through anonymised electronic routine medical records via CPRD. The ease of the study, assignment of recruitment responsibility efforts to the research team, including input from the academic GP collaborator, may have been a contributing factor in achieving the recruitment target. The use of a ‘discipline champion’ in aiding recruitment has also been previously reported.^22^

As practices were predominantly recruited from CPRD, this could impact on the generalisability of results. That said, some of the practices in CPRD (*n*=37) had not previously participated in RCTs before PLEASANT. At the time of recruitment, only practices using the Vision system were able to contribute to CPRD, so this reduced potential participation from practice using either TPP SystmOne or EMIS (Leeds, UK), the other two major GP computer systems in the United Kingdom. However, CPRD’s practice populations have been shown to be representative of the UK GP population, the exception being a deficit of children aged 0–4 years and an excess of patients aged 85 years and over^23^ neither of which are the population of interest for the PLEASANT trial. CPRD are currently working to include TPP SystmOne and EMIS that will broaden opportunities for practice participation in the future.

### Implications for future research, policy and practice

If more experienced practices are likely to participate in future studies, this would suggest time and investment is needed to engage research naive settings. Previous studies have indicated that the importance of the research question,^4,21,24–28^ the need for simple studies that are not time consuming^4,24,25^ and that do not affect the doctor–patient relationship^4,25,27^ may be the way to encourage practice participation. The PLEASANT trial was designed to reduce the research burden as much as possible and this may have contributed to recruitment. A side product of doing PLEASANT is that it has also increased the number of practices in CPRD that have now participated in an RCT that would hopefully be more likely to participate in future research.

Researchers need to consider the amount of resource and potential strategies required to maximise recruitment as one method alone may not produce the outcomes required.

### Conclusions

Recruitment to time and target is achievable though the amount of resource required for site recruitment should not be underestimated. The ease of the study, good communication, an understanding of the context and environment of practice, and the experience of sites may have some bearing on practice participation and retention in primary care clinical trials. General practices with more research experience are more likely to participate in RCTs.

## Figures and Tables

**Table 1:** Odds ratios, 95% confidence intervals and P-values for logistic regression models

*Dependent variable*	*Independent variable*	*OR (95% CI)*	P-*value*
EOI	Number of previous studies	1.98 (1.57–2.51)	<0.001
	Number of individual RCTs	2.51 (1.71–3.78)	<0.001
	Number of cluster RCTs	2.16 (1.57–2.99)	<0.001
	Practice size (1,000's)	1.03 (0.99–1.08)	0.160
			
Randomised	Number of previous studies	1.91 (1.52–2.42)	<0.001
	Number of individual RCTs	2.42 (1.65–3.61)	<0.001
	Number of cluster RCTs	2.07 (1.51–2.86)	<0.001
	Practice size (1,000's)	1.04 (0.99–1.08)	0.137

Abbreviations: CI, confidence interval; EOI, expression of interest; OR, odds ratio; RCT, randomised controlled trial.

**Table 2:** Hazard ratios, 95% confidence intervals and P-values for Cox proportional hazard models

*Dependent variable*	*Independent variable*	*HR (95% CI)*	P-*value*
EOI	Number of RCTs	1.83 (1.58–2.12)	<0.001
	Number of individual RCTs	2.36 (1.86–2.99)	<0.001
	Number of cluster RCTs	1.91 (1.52–2.40)	<0.001
	Practice size (1,000's)	1.03 (0.99–1.07)	0.122
			
Randomised	Number of RCTs	1.81 (1.55–2.11)	<0.001
	Number of individual RCTs	2.36 (1.85–3.02)	<0.001
	Number of cluster RCTs	1.83 (1.45–2.32)	<0.001
	Practice size (1,000's)	1.04 (0.10–1.08)	0.054

Abbreviations: CI, confidence interval; EOI, expression of interest; OR, odds ratio; RCT, randomised controlled trial.
